# Hard X-ray Fourier transform holography from an array of oriented referenced objects

**DOI:** 10.1107/S0909049511009836

**Published:** 2011-05-11

**Authors:** Hiroyuki Iwamoto, Naoto Yagi

**Affiliations:** a Research and Utilization Division, SPring-8, Japan Synchrotron Radiation Research Institute, Hyogo 679-5198, Japan

**Keywords:** Fourier transform holography, hard X-rays, X-ray bandwidth, X-ray microbeam

## Abstract

Hard X-ray Fourier transform holography (HXFTH) is a promising method for imaging nanoscale objects, including biological molecules, with a spatial resolution of a nanometer or better. However, it suffers from low scattering intensities being available for imaging owing to smaller object size and the low scattering cross section inherent in hard X-rays. One technique to overcome the problem would be to use an array of oriented objects, each with its own reference. Here the feasibility of this approach was experimentally tested by recording diffraction patterns from nanofabricated test patterns arranged in a 5 × 5 matrix. At an X-ray energy of 8 keV (λ = 1.55 Å), the image of the original test pattern was clearly restored with 60 s exposure on an imaging plate; the image was still recognizable with a 500 ms exposure on a CCD detector at the BL40XU beamline at SPring-8. The results demonstrate that the use of an array of referenced oriented objects for HXFTH is workable, and that it can be considered as a practical candidate for imaging biological molecules, identical particles of which are available but diffract even more weakly than artificially fabricated test patterns.

## Abstract 2

The use of multiple, oriented and singly referenced objects was evaluated as a means to overcome the low scattering cross section inherent in hard X-ray Fourier transform holography. It is shown that the image of each object can be restored as in the conventional single-object case.

## 1. Introduction

The use of hard X-rays in imaging nanoscale objects is expected to provide atomic resolution owing to its short wavelengths. A major obstacle in achieving this goal has been the phase problem, *i.e.* the phase information needed to restore the original structure image is lost when scattering from the sample is recorded on the detector. Recently, however, a number of methods to restore the original structure image have been devised by retrieving phase information from diffraction/scattering patterns. One is the oversampling method (Miao *et al.*, 1999 ▶), which does not require references but requires good photon-counting statistics and a large number of iterative calculations. The other is X-ray Fourier transform holography (FTH; McNulty *et al.*, 1992 ▶; Eisebitt *et al.*, 2004 ▶; He *et al.*, 2004 ▶; Schlotter *et al.*, 2006 ▶, 2007 ▶; Mancuso *et al.*, 2010 ▶). Although at least one reference point object must be placed near the sample, the principle is straightforward and the image of the original structure can be restored with a single Fourier transform operation. Unlike the oversampling method, FTH is apparently resistant to low photon-counting statistics (Schlotter *et al.*, 2006 ▶). The size of the reference dot is a major factor in defining the spatial resolution. Reducing the reference size increases the spatial resolution, but this reduces the intensity of the reference wave needed to restore the original structure. Thus, there is a tradeoff between resolution and signal strength and this can pose a major limitation for FTH.

X-ray FTH was first successfully tested in the soft X-ray regime and the spatial resolution obtained was ∼50 nm. If the same technique is extended to the hard X-ray regime, the spatial resolution will be improved accordingly and hard X-ray FTH (HXFTH) may serve as an alternative structure-analysis technique that can compete with electron microscopy for spatial resolution. The first HXFTH study has been made by utilizing the focus of a Fresnel zone plate as a reference spot (Watanabe *et al.*, 2003 ▶; see also McNulty *et al.*, 1992 ▶ for technique), and later by using a lithographically fabricated test pattern with built-in reference dots (Stadler *et al.*, 2008 ▶).

The problem encountered by HXFTH is the reduced scattering intensities available for image restoration. Three factors contribute to this reduction:

(i) reduced scattering cross section owing to increased X-ray energy,
(ii) reduced sample size (it is the purpose of HXFTH to image smaller objects), and
(iii) demand to reduce reference size to increase spatial resolution, resulting in a reduced sample–reference interference.

A few methods to increase scattering intensities have been devised. One is to increase the number of references (Schlotter *et al.*, 2006 ▶; Stadler *et al.*, 2008 ▶). In this case, an odd number of references are placed around the sample. This increases the number of reconstructed images accordingly and the signal-to-noise ratio is improved by averaging the images. However, too many references would result in overlapped images, and the maximal number of references (= intensity gain) is practically limited to five. A more sophisticated method to increase scattering intensities is the use of a large complex-shaped reference with deconvolution of the resulting image (He *et al.*, 2004 ▶; Marchesini *et al.*, 2008 ▶). By using this technique the efficiency of holography can be significantly improved. In applying this technique for HXFTH it would be a major issue how to fabricate such a complex reference with a smaller physical dimension suitable for hard X-ray imaging.

Here we consider a third approach to increase scattering intensities, *i.e.* to increase the number of samples, each with its own reference(s). As each sample has its own reference, one should be able to increase the strength of the sample–reference interference without sacrificing spatial resolution as opposed to the use of a larger reference which generates a stronger reference wave. The conditions for applying this method are that:

(i) a number of identical samples are available, and
(ii) they can be oriented with respect to the reference.

In the case of biological nanoparticles (such as protein molecules and their assemblies) each particle scatters X-rays very weakly; it would be the only way to use a large number of particles and to orient them in order to apply HXFTH to this category of samples.

In this study we tested the feasibility of this approach by recording diffraction patterns from singly referenced identical nanopatterns arranged in a 5 × 5 matrix by using X-ray microbeams at 8 keV (λ = 1.55 Å). The results demonstrate that the image of the sample can be restored as in the conventional single-sample case without the need to accumulate a large number of exposure frames.

## 2. Materials and methods

### 2.1. Optics

**Figure 1:**
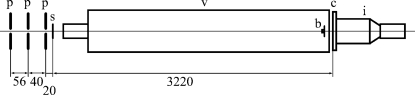
Schematic diagram of X-ray optics used in the study. Upstream left; b: beamstop and aluminium mask; c: cassette for imaging plate, which allows the use of the CCD camera behind when the plate is removed; i: image intensifier and CCD camera; p: pinholes made of 50 µm tantalum. The aperture sizes are 10, 20 and 50 µm from upstream. s: sample on a motorized *x*–*z* stage; v: vacuum path. Dimensions are in mm.

Diffraction patterns were recorded at the BL40XU beamline of SPring-8, Japan (Inoue *et al.*, 2000 ▶). Partial beam coherence was obtained from the 8 keV X-ray beam (λ = 1.55 Å) through a 10 µm defining pinhole (50 µm-thick tantalum), followed by two guard pinholes. The distance between the defining pinhole and the sample was 116 mm. The beam was attenuated to 1/100 by a fast-rotating disc shutter. The flux after attenuation was estimated to be ∼10^11^ photons s^−1^. A long vacuum path was placed between the sample and the detector, and the specimen-to-detector distance was 3.2 m. Immediately upstream of the detector was placed a beamstop (Ø = 2 mm) and a round aluminium foil attenuator (Ø 6 mm × 100 µm and Ø 20 mm × 50 µm) to compensate for the detector dynamic range. The sample was aligned with the beam by monitoring with a cooled CCD camera (C-4880, Hamamatsu Photonics) in combination with an image intensifier (V5445P-MOD, Hamamatsu Photonics). Its pixel size was 150 µm. After alignment, diffraction patterns were recorded on imaging plates (Fuji Film) and were read by an imaging-plate reader (BAS-2500, Fuji Film) with 50 µm pixel size. After recording the diffraction from the test pattern the sample was moved so that the beam hit the plain gold foil, the scattering from which was later subtracted from the diffraction from the test pattern. A schematic diagram of the optics layout is shown in Fig. 1 ▶.

### 2.2. Sample

**Figure 2:**
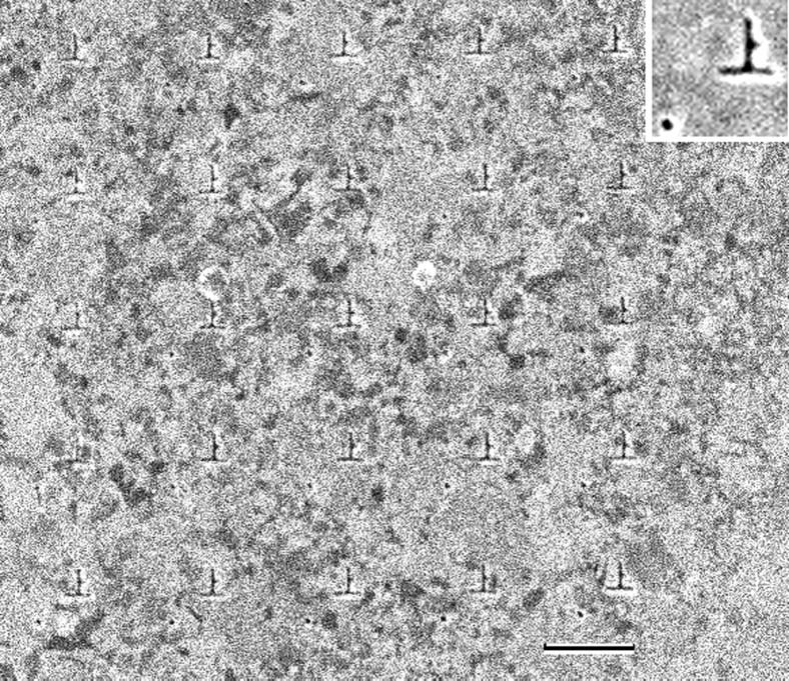
Scanning ion micrograph of the test pattern fabricated on 0.5 µm gold foil. Chinese characters meaning ‘up’ are arranged in a 5 × 5 matrix. Each character has its own reference dot on the lower left. Scale bar: 1 µm. Inset: average of the images of 25 characters (2× magnification).

The test pattern consisting of 25 pairs of a letter (Chinese character meaning ‘up’) and a dot, arranged in a 5 × 5 square matrix, was fabricated by focused ion-beam abrasion on 0.5 µm gold foil (custom-made by Seiko I Techno Research, Japan; Fig. 2 ▶). The letter was ∼0.26 µm (width) × 0.28 µm (height), and the dot (diameter ∼45 nm) was 0.41 µm from the lower edge of the letter. The pitch of the matrix was ∼1.5 µm.

### 2.3. Data processing

The image of the test pattern was restored by calculating the Fourier transform of the diffraction pattern by setting all the phases to zero (Patterson map) after calculating the square root of the intensities. The Patterson maps from the original diffraction patterns gave rise to false densities where densities should not exist, because some of the intensities are lost owing to beamstop and parasitic scattering from the pinholes. To minimize false densities, the lost intensities under the beamstop were restored by repeated Fourier transformation followed by Patterson map calculation. The intensities outside the beamstop were left unchanged (see He *et al.*, 2004 ▶). This procedure does not contain a randomizing operation, and the filling pattern under the beamstop is uniquely determined.

The effect of missing pixels on the quality of the restored image has been discussed in the case of oversampled diffractions (Miao *et al.*, 2005 ▶). They argued that some structural information is permanently lost when the size of missing data is greater than a single speckle size, equivalent to the inverse of the sample size. In the case of FTH, the speckle size is scaled to the inverse of the specimen-to-reference distance, which is smaller than the inverse of the sample size itself. Thus, the restriction is more severe for FTH than the oversampling method. In our case the beamstop diameter corresponds to 1/500 nm^−1^, slightly smaller than the speckle size (∼1/300 nm^−1^). A greater beam size would cause the distortion of restored images as well as false densities.

## 3. Results and discussion

### 3.1. High-resolution diffraction pattern

**Figure 3:**
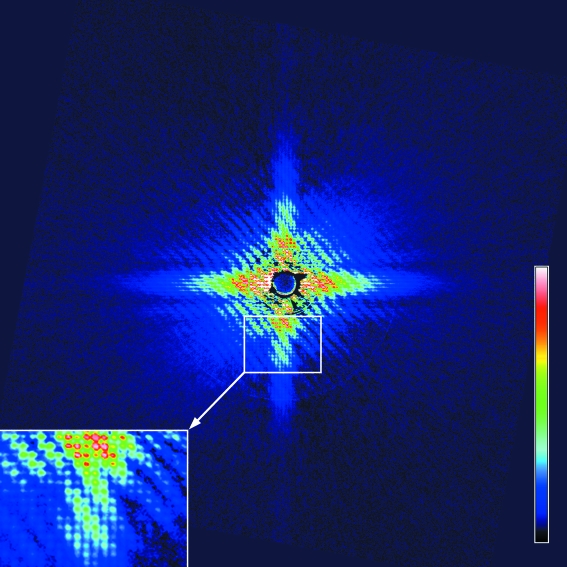
Pseudocolor representation of the diffraction pattern (hologram) of the test pattern shown in Fig. 2 ▶, recorded on an imaging plate (exposure, 1 min; pixel size, 50 µm). Scattering from the plain gold foil has been subtracted and the diffraction pattern has been square-rooted to indicate the true amplitude of the structure factor. The inset on the lower left shows a magnified view of part of the diffraction pattern, showing the sampling owing to the 5 × 5 matrix. The patterns have been rotated by 10.6° to match the lattice orientation, as the lattice of the sample was not visible at the time of mounting.

The diffraction pattern (hologram) recorded on an imaging plate (50 µm resolution) is shown in Fig. 3 ▶. Besides the moiré pattern arising from the interference between the letter and the reference dot, the entire pattern is finely sampled into a square lattice (inset in Fig. 3 ▶). Since the lattice constant is 1.5 µm, this indicates that the transverse coherence is extended over a micrometer-scale area.

**Figure 4:**
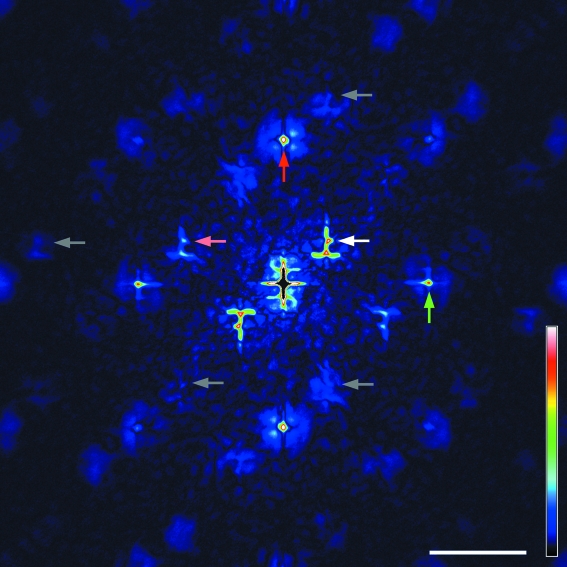
Pseudocolor representation of the Fourier transform (Patterson map) of the diffraction pattern as shown in Fig. 3 ▶, after restoration of the intensities under the beamstop (see §2). Besides the pair of images of the Chinese character around the center (white arrow), additional images were created in the outer area owing to the matrix arrangement of characters. Of these only a single pair is clearly recognized (pink arrow). Others are corrupt (gray arrows). These arrows point to the short sidebar of the Chinese character. The upward pointing arrows indicate the centers of additional autocorrelation terms. Note that the vertical ones (red arrow) are brighter than the horizontal ones (green arrow). Scale bar: 1 µm.

The Fourier transform of the diffraction pattern shown in Fig. 3 ▶ (Patterson map) generates a pair of restored images of the letter on both sides of the central autocorrelation terms, as expected from a single pair of sample and reference (Fig. 4 ▶). Since the sample–dot interference is recognized for up to 15th order, or 1/40 nm^−1^, the spatial resolution of the hologram is considered to be 40 nm. The restored image of the letter, or the cross-correlation term between the letter and the reference dot, is somewhat distorted and some nonuniformity is observed in its density. This could be due to partial coherence of the beam (Stadler *et al.*, 2008 ▶).

As expected from the arrangement of the test patterns in a two-dimensional matrix, additional sets of restored images and the central autocorrelation are regularly arranged in the Patterson map. In an ideal situation (the matrix is completely regular and extends infinitely, and the beam has infinite coherence) the sets should also be arranged in the shape of a matrix, and an infinite number of images would be restored. In the map shown in Fig. 4 ▶, however, these extra images become fainter as they are positioned further away from the center. The images also become corrupt, and only one additional pair of letters are clearly recognized. This deterioration may be due to the limited extent of the two-dimensional matrix, partial coherence and also the wide bandwidth of the X-ray beam, as discussed later.

### 3.2. Low-resolution diffraction pattern

**Figure 5:**
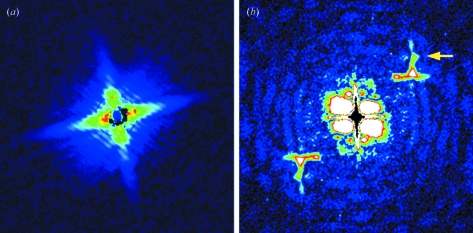
Diffraction pattern and Fourier transform of the same sample as in Figs. 3 ▶ and 4 ▶, but recorded on a CCD detector (exposure 500 ms; pixel size 150 µm). (*a*) Diffraction pattern; (*b*) Fourier transform. In (*b*) a pair of character images are recognized, with somewhat inferior quality (yellow arrow).

In this study a CCD camera (150 µm resolution) was used for monitoring purposes, but diffraction patterns were also recorded for the analyses (Fig. 5 ▶ *a*). We tested whether these diffraction patterns yielded restored images upon Patterson map calculation.

Owing to the lower resolution of the detector the lattice sampling of the pattern was not resolved, but the interference between the letter and the dot was clearly recognized with a single 500 ms exposure. In the Patterson map (Fig. 5 ▶ *b*) a pair of restored images of the letter can be clearly recognized, although they are noisier and the upper part of the image does not have enough density. With a short exposure of 500 ms, the quality of the restored image was also affected by Poisson noise in the diffraction pattern. The peak intensity in the diffraction pattern was ∼10000 counts (AD converter output), while the noise level was ∼60 counts. Therefore, the signal-to-noise ratio was only of the order of 100, but the image was nevertheless restored.

The restoration of images with such a low signal-to-noise ratio and low-resolution exposure demonstrates the robustness of the analysis, as well as the effect of the array of samples in increasing scattering intensity. The observed ability to restore images is also remarkable considering that the scattering from Au at 8 keV is much weaker than at conventionally used soft X-ray energies, typically ∼800 eV.

### 3.3. Effect of wider energy bandwidth of X-ray beam

The BL40XU beamline is designed to utilize the fundamental wave of a helical undulator to obtain quasi-monochromatic X-ray beams, instead of using a monochromator. The bandwidth of the X-ray beam (Δλ/λ) is 1.6% (full width at half-maximum) as opposed to 0.01% in beamlines equipped with double-crystal monochromators. Owing to this wide bandwidth, the BL40XU beamline attains a photon flux of 7 × 10^14^ s^−1^ at 8 keV. For FTH applications, however, the wide bandwidth limits the resolution in restored images. This is because higher-order reflections from any set or array of objects start to overlap with each other. In the case of ∼2% bandwidths, reflections are not expected beyond ∼30th order depending on the sharpness of each reflection peak. This means that the sample–dot interference needed for image restoration will not be obtained beyond these orders. In the present case the distance between the center of gravity of the sample and the reference dot is ∼600 nm so that the resolution limit will be ∼20 nm (1/30 of 600 nm).

Likewise, the lattice sampling originating from the matrix is also expected to disappear beyond ∼30th. Up to the 27th peak, corresponding to ∼56 nm, is recognized in Fig. 3 ▶. This means that the lattice interference diminishes faster than the sample–dot interference, and that only lower-resolution information is available to restore extra images in the outer area of the Fourier transform. This partly explains why the images restored in the outer area are corrupt (see Fig. 4 ▶).

The wide bandwidth could also affect the beam coherence. The longitudinal coherence, defined as λ^2^/Δλ, is only ∼10 nm here. Nevertheless the image is successfully restored, indicating that the transverse coherence is more important for FTH to be successful. If the defining pinhole works as a point light source, the transverse coherence length at the sample is isotropic and is a function of wavelength, pinhole size and the pinhole–specimen distance (∼1.9 µm in the current settings). However, the clear lattice sampling suggests that the actual coherence length is better than this value, indicating that the components upstream of the pinhole define the coherence length. In fact, in the Patterson map in Fig. 4 ▶ the centers of additional autocorrelation terms (upward-pointing arrows) are brighter in the vertical direction (red) than in the horizontal direction (green), reflecting the anisotropy of the original beam in which the vertical coherence is better than the horizontal coherence.

Regardless of all the considerations above, the sample–dot interference (which requires a shorter coherence length) lasted only up to 15th order as opposed to 27th order in the lattice interference. This means that the X-ray bandwidth or the partial coherence is not a primary factor to limit spatial resolution to restore the image. Rather, it is likely to be the reference size that determines the resolution. Although the coherence length here may not be long enough for all the 25 members of the lattice to diffract coherently, each sample–reference pair should diffract coherently and their intensity should sum together in the recorded diffraction pattern. With X-ray bandwidth unchanged, the resolution will be further improved if the three parameters (sample size, reference size and sample-to-reference distance) are proportionally scaled down. This operation will also make the sample–reference pair much shorter than the coherence length. In future high-resolution studies with smaller references with respect to the sample, however, high monochromaticity will be a prerequisite.

### 3.4. Conclusion and prospects

In this study the use of an array of oriented individually referenced samples has been evaluated as a means of compensating for the low-scattering intensities in HXFTH. The Patterson map from the diffraction pattern of a 5 × 5 array of referenced objects restored an image of the objects with a spatial resolution comparable with that published for single objects, demonstrating the validity of the method. Arranging the objects in a regular two-dimensional array has the extra benefit of creating multiple images available for averaging, but the deviation from ideal conditions (lattice imperfectness, wide X-ray bandwidth and partial coherence) causes peripheral images to corrupt.

The use of an array of samples would be especially useful for weakly scattering biological particles (such as protein molecules and their assemblies). The molecules may be fitted into a self-assembled two-dimensional lattice with a heavy-atom reference particle in each lattice point. Alternatively, HXFTH may be applied while the molecules are in solution. In this case each molecule is labeled with its own reference, such as a gold cluster. These molecules may then be oriented two-dimensionally by applying two different forces at the same time, *e.g.* shear-flow (Sugiyama *et al.*, 2009 ▶) and electric field. Currently, small-angle scattering of protein solutions, in which molecules are randomly oriented, is utilized for the *ab initio* determination of low-resolution three-dimensional structures (*e.g.* Svergun, 1999 ▶). *Ab initio* structure determination usually involves iterative procedures based on genetic algorithms, simulated annealing *etc.*, and its solutions face a uniqueness problem. If the technical issues of labeling with reference particles and orienting the molecule are resolved, HXFTH can be a good alternative structure determination method because of its ability to yield unique solutions with a single-step Fourier transformation.

Finally, it should be emphasized that the hard X-rays from the third-generation synchrotrons have enough local coherence to illuminate individual small sample–reference pairs coherently (this property has already been utilized for conventional diffraction/scattering studies). It is expected, therefore, that the full flux of the monochromated beam (without the need to use pinholes) can be used for multi-object HXFTH imaging of biological molecules.
